# Construction of Cohorts of Similar Patients From Automatic Extraction of Medical Concepts: Phenotype Extraction Study

**DOI:** 10.2196/42379

**Published:** 2022-12-19

**Authors:** Christel Gérardin, Arthur Mageau, Arsène Mékinian, Xavier Tannier, Fabrice Carrat

**Affiliations:** 1 Institute Pierre Louis Epidemiology and Public Health Institut National de la Santé et de la Recherche Médicale, Sorbonne Université Paris France; 2 Institut National de la Santé et de la Recherche Médicale Unité Mixte de Recherche 1137 Infection Antimicrobials Modelling Evolution, Team Decision Sciences in Infectious Diseases Université Paris Cité Paris France; 3 Service de Médecine Interne, Inflammation-Immunopathology-Biotherapy Department Hôpital Saint-Antoine, Sorbonne Université Assistance Publique–Hôpitaux de Paris Paris France; 4 Laboratoire d'Informatique Médicale et d'Ingénierie des Connaissances pour la e-Santé Institut National de la Santé et de la Recherche Médicale, Université Sorbonne Paris France; 5 Public Health Department Hopital Saint-Antoine Assistance Publique–Hôpitaux de Paris Paris France

**Keywords:** natural language processing, similar patient cohort, phenotype, systemic disease, NLP, algorithm, automatic extraction, automated extraction, named entity, MeSH, medical subject heading, data extraction, text extraction

## Abstract

**Background:**

Reliable and interpretable automatic extraction of clinical phenotypes from large electronic medical record databases remains a challenge, especially in a language other than English.

**Objective:**

We aimed to provide an automated end-to-end extraction of cohorts of similar patients from electronic health records for systemic diseases.

**Methods:**

Our multistep algorithm includes a named-entity recognition step, a multilabel classification using medical subject headings ontology, and the computation of patient similarity. A selection of cohorts of similar patients on a priori annotated phenotypes was performed. Six phenotypes were selected for their clinical significance: P1, osteoporosis; P2, nephritis in systemic erythematosus lupus; P3, interstitial lung disease in systemic sclerosis; P4, lung infection; P5, obstetric antiphospholipid syndrome; and P6, Takayasu arteritis. We used a training set of 151 clinical notes and an independent validation set of 256 clinical notes, with annotated phenotypes, both extracted from the Assistance Publique-Hôpitaux de Paris data warehouse. We evaluated the precision of the 3 patients closest to the index patient for each phenotype with precision-at-3 and recall and average precision.

**Results:**

For P1-P4, the precision-at-3 ranged from 0.85 (95% CI 0.75-0.95) to 0.99 (95% CI 0.98-1), the recall ranged from 0.53 (95% CI 0.50-0.55) to 0.83 (95% CI 0.81-0.84), and the average precision ranged from 0.58 (95% CI 0.54-0.62) to 0.88 (95% CI 0.85-0.90). P5-P6 phenotypes could not be analyzed due to the limited number of phenotypes.

**Conclusions:**

Using a method close to clinical reasoning, we built a scalable and interpretable end-to-end algorithm for extracting cohorts of similar patients.

## Introduction

### Background

Extracting clinical phenotypes from large electronic health record (EHR) databases, also known as clinical data warehouses, is a key step for several medical applications from epidemiological research [1] to prognosis prediction [2,3] and therapeutic decision support [4,5]. Reliable automatic extraction of patient phenotypes from large EHR databases remains a challenge, especially in languages other than English [6]. The actual identification of patients’ phenotypes is still largely done via the International Classification of Diseases, Ninth/Tenth Revision (ICD-9/ICD-10) code extraction, reading of clinical notes, or extraction of entities via regular expressions. However, as shown by Farzandipour et al [7] on more than 300 EHR ICD-10 codes, 22.7% presented errors in principal diagnosis codes, of which 33.3% were major errors. Benkhaial et al [8] also showed in a study of 200 patients, ICD allergy codes were present for 18 patients, while 51 had allergy information in a written note, indicating that only 35% of the allergies were correctly coded. These identification methods thus lack precision and require important human control.

With the improvement of natural language processing over the last 10 years, new language models such as Word2vec [9], GloVe [10], FastText [11] and, more recently, Bidirectional Encoder Representations from Transformers (BERT) [12] have allowed significant progress for various natural language processing tasks such as translation, question-answering, and named-entity recognition via an efficient word representation. Named-entity recognition corresponds to the extraction of certain classes of entities in a raw text. In the medical domain, it can be “signs and symptoms,” “disorders,” “chemicals and drugs,” etc.

Many research teams have developed new algorithms based on these word models to allow automatic patient phenotyping. De Freitas et al [13] proposed Phe2vec, a data-driven, unsupervised disease phenotyping algorithm. In their study, disease phenotypes correspond to the word representation of ICD-10 core concepts (or seed concepts) and their closest neighbors. A patient’s clinical history is summarized by aggregating all the word vector representations of the medical concepts. Mapping a patient to a disease is then done by computing a cosine distance between the patient with each disease phenotype. In their method, the medical concept extraction step from clinical notes is performed based on 1 ontology [14]. Ferté et al [15] also proposed an algorithm for automatic phenotyping of EHRs by using ICD-10 codes and a dictionary-based entity recognition tool to extract interesting terms from clinical notes. Extracted terms were then mapped to their unified medical language system concept unique identifier as a feature for classification to provide an interpretable parametric predictor. Their work showed particularly interesting results for chronic conditions.

In this work, we extracted similar patients by focusing on 4 systemic diseases as a proof of concept: systemic lupus erythematosus (SLE), systemic sclerosis, antiphospholipid syndrome (APS), and Takayasu arteritis. SLE is an autoimmune disease that can affect a large number of organs: the skin (specific malar rash, photosensitivity, etc), kidneys (nephrotic syndrome and glomerular nephropathy), joints (most often without deformation), brain (with neuropsychiatric forms), etc. It is a rare disease that affects 41 in 100,000 people in France [16], and 9 women for 1 man in generally young (18-30 years old) adults. Systemic sclerosis can also involve various organs: the skin (sclerosis leading to significant functional impotence), the lungs (interstitial lung disease [ILD], fibrosis, and hypertension), the digestive system (reflux and chronic intestinal obstruction), etc. Its frequency is 1/5000 in France, and it preferentially affects women (4 women for 1 man) aged between 40 and 50 years. APS is a disease that causes venous and arterial thrombosis as well as obstetrical complications. Approximately 20%-30% of patients with lupus develop APS. Its frequency is approximately 1 in 12,000 [16]. Takayasu arteritis is an inflammatory disease that affects large vessels in young people. It is a very rare disease affecting 1.2 to 2.6 cases/million/year in France. It affects 4.8 women for 1 man between 20 and 40 years of age [17]. These 4 diseases were chosen because of their large spectrum of signs and symptoms and their similarity (especially for lupus and APS in terms of apparition frequency and APS and Takayasu for their arterial manifestations).

### Goal of This Study

In this study, we aimed to develop an automated end-to-end extraction of similar patient cohorts from electronic medical records. Specifically, we place ourselves in the following use case: we have a patient to treat with clinical information in a text document (mentioned as index patient in this paper), and we automatically search for the set of patients with similar symptoms and diseases mentioned in their hospitalization reports. To evaluate our method, we extracted cohorts of similar patients from index patients with certain phenotypes described in their textual reports, arbitrarily selected, and manually annotated by a clinician. Our main contribution in this paper is the development of an algorithm for the automatic construction of similar patient cohorts by a method close to clinical reasoning, as we argue in the Discussion section.

## Methods

### Algorithm Steps

In this section, we detail the main steps of our algorithm. Similarity is defined here as a patient with identical or closely related signs, symptoms, and disorders. The key steps for extracting these events from the text are a named-entity recognition step to extract medical concepts, a multilabel classification on each extracted term, and an average distance computation on an appropriate representation of all the terms on each label. We validated our interpatient distance by clustering 6 a priori defined phenotypes of interest: osteoporosis, nephritis in SLE, ILD in systemic sclerosis, lung infection, obstetric APS, and Takayasu arteritis. With the same interpatient distance, we then constructed similarity cohorts from index patients for each of these phenotypes.

### Overview of the Algorithm

For readability, in the remainder of this paper, we use the term “patient” to refer to the “hospitalization report related to the patient.”

**Figure 1:**
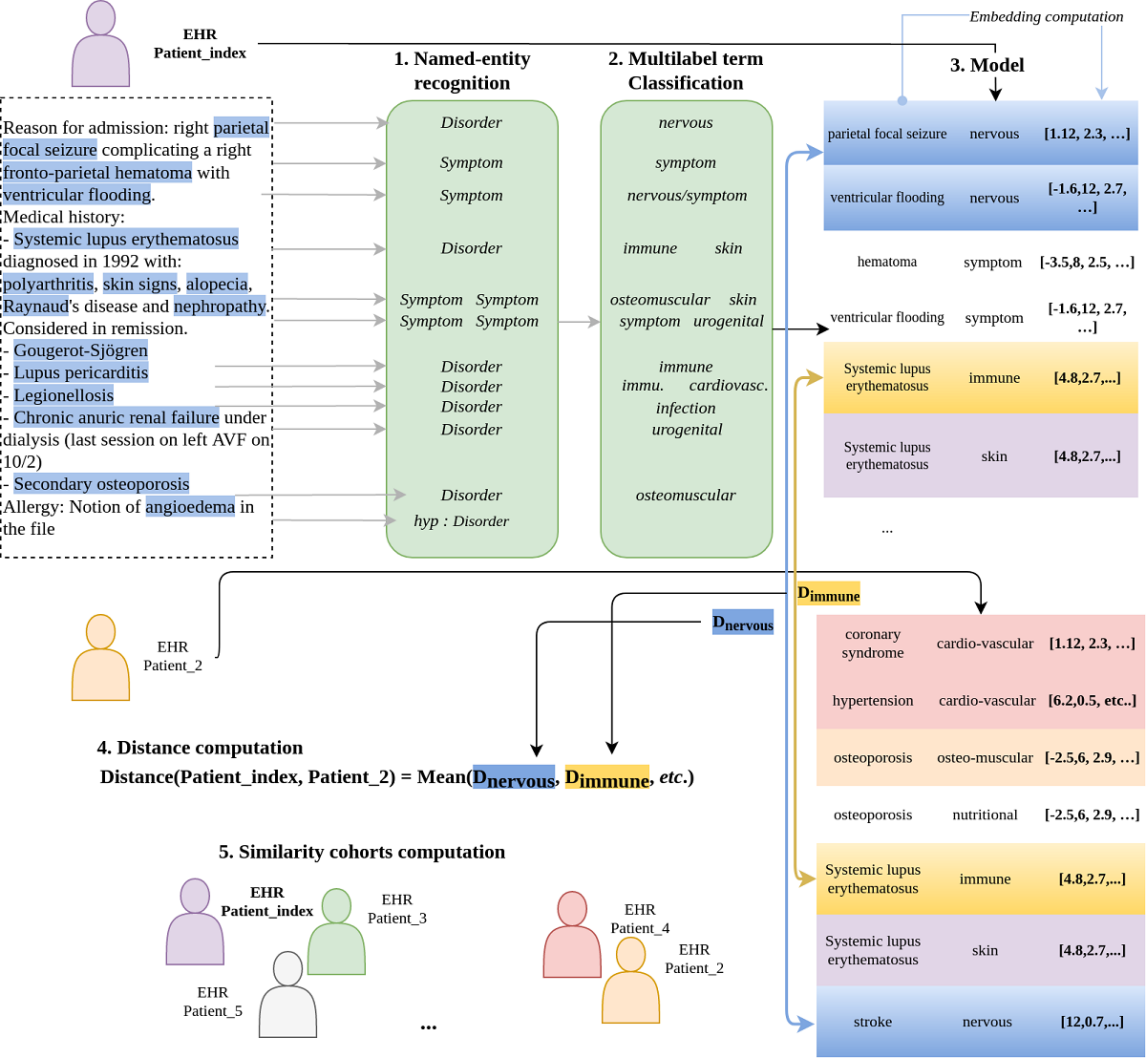
Overview of the algorithm to obtain a representation of the patients’ electronic health records and to compute a distance from other patients’ electronic health records. First, a named-entity recognition step is performed on a patient's electronic health record (to extract symptoms and disorders and filter all negated, hypothetical, and someone else’s terms). Second, a multilabel classification step is performed for each extracted term to allow more clinical interpretation. Third, this leads to an electronic health record model containing all the extracted terms with their respective labels and embedding representations (last column of the model). Fourth, this allows a distance computation on each of the 22 labels (Dnervous corresponds to the distance between embeddings of all terms labelled nervous, Dimmune on the immune label, etc). Fifth, a similarity cohort computation is performed. EHR: Electronic Health Record.

The main steps of the algorithms are shown in Figure 1, considering an index patient:

1. Symptoms and diseases were extracted from a raw text while filtering out all negated, hypothetical, and belonging to family terms.
2. All extracted terms were classified into broad organ categories, that is, cardiovascular, immune, ophthalmologic, digestive, etc, by a multilabel classification step using our previously developed algorithm [18].
3. A vector (embedding) representation for all extracted terms was obtained leading to the index patient representation.
4. From this representation for other patients, the distance for each label of the index patient to the other patients was computed. Then, the average of the distances of all the labels was determined.
5. A cohort of similar patients was built from the patients closest to the index patient for each annotated phenotype.

We will refer to this patient’s hospitalization report (Figure 1, index_patient) as a running example throughout the steps described below.

### Data Sets and Annotation Rules

The data set of this study was obtained from the Assistance Publique-Hôpitaux de Paris (AP-HP) data warehouse. Patients were informed that their EHR information could be reused after an anonymization process, and those who objected to the reuse of their data were excluded. All methods were carried out in accordance with relevant guidelines (reference methodology MR-004 of the Commission Nationale de l’Informatique et des Libertés [19]).

The data set contained all hospitalization reports, consultation reports, test results, prescriptions, etc of all patients older than 15 years with lupus, scleroderma, APS, and Takayasu arteritis who made at least one visit to AP-HP hospitals since 2017. The data set constitutes a set of 2 million pseudonymized clinical records. It was extracted using only the ICD-10 codes of the principal diagnosis for lupus (M320, M321, M328, M329, L930, L931, corresponding to 5176 patients), systemic sclerosis (M340, M341, M348, M349, corresponding to 2833 patients), APS (D686 corresponding to 1250 patients), and Takayasu arteritis (M314, corresponding to 287 patients).

An internist physician annotated a training subset of 151 clinical notes (40 lupus, 35 APS, 37 systemic sclerosis, and 39 Takayasu) with symptoms or disorders by using specific attributes “negated,” “hypothetical,” and “belonging to family” when relevant. Guided by a clinical logic, we chose not only to annotate the negated terms as negation (eg, no fever, no diabetes) but also all the physiological descriptions (eg, peripheral pulse present, vesicular breath sounds present and symmetrical, regular heart sounds). All of these physiological findings were annotated as negative, because in clinical reasoning, we focus primarily on pathological signs. We adopted this approach also because the language models we use are able to capture both the syntactic and semantic levels of language. The medical subject heading (MeSH) category C [20] head chapters (eg, cardiovascular, immune, digestive) were also annotated at the entity level. This annotated data set was used to train both the named-entity recognition step with the symptoms and disorders labels and the multilabel classification step with MeSH [20] category C chapter head labels. Another test set of 256 hospitalization reports was annotated with one or more of the 6 phenotypes of interest, that is, osteoporosis, nephritis in SLE, ILD in systemic sclerosis, lung infection, obstetric APS, and Takayasu arteritis by another internist physician with no common patients between the training and testing data sets.

The annotation rules were defined before starting. First, a phenotype was only positively annotated if it was explicitly written, and no interpretation was made of signs and test results to guess the phenotype. For example, for osteoporosis, neither bone mineral density nor the number of vertebral fractures was interpreted, and the only terms retained positively were osteoporosis and corticosteroid-induced osteoporosis. Detailed examples can be found in Figure S1 of Multimedia Appendix 1. We selected these phenotypes for their clinical significance both in the 4 pathologies of interest studied and globally in terms of osteoporosis and lung infection phenotypes. These phenotypes were selected as an example, but our algorithm can be generalized to handle very different phenotypes.

### Word Representations

Two word representation models were used for this work. First, a French BERT model [12], camemBERT, trained by Martin et al [21] on a wide variety of French documents was used for the named-entity recognition and multilabel classification steps. Second, a FastText model developed by Bojanowski et al [11] was used for the patient model to calculate the interpatient distance. Both methods convert words into vectors of real numbers (called embeddings). BERT produces embeddings that take into account the context (other words in the phase), while FastText produces fixed embeddings (a word corresponds to a vector independently of the surrounding text). For our study, we had 2 million documents of all types (consultation records, hospitalization records, discharge summaries, etc), which correspond to a volume of 5 gigabytes of text. These data allowed us to train the FastText model from scratch. The camemBERT model was too large to train from scratch, but we fine-tuned it on our data, that is, we retrained its final layers. As a result, it was able to learn a context-appropriate vector representation (particularly effective for the feature extraction step 1); nevertheless, its initial vocabulary did not contain all the medical concepts, unlike the FastText model, which we used for the patient representation for the interpatient distance calculation.

### Named-entity Recognition

This first step enables us to extract positive symptoms (pathologic signs) and disorders, filtering all terms corresponding to hypothetical, negated, and family-related elements. For instance, in Figure 1 (index_patient), the extracted terms were “parietal focal status epilepticus,” “frontoparietal hematoma,” and “systemic lupus erythematosus,” whereas “angioedema” was not kept since it was only hypothetical. The algorithm used for this first step is based on an encoder (with BERT layers) and a bidirectional long short-term memory decoder. This neural named-entity recognition model, described in [18], obtains an exact F-measurement of 0.931 on the English CoNLL data set [22], using the BERT-large embeddings [12], and 0.784 on GENIA [23], using the BioBERT-large model [24].

### Multilabel Classification

To improve clinical interpretability and to analyze patients along several medical dimensions (ie, labels), we chose to perform a multilabel classification of all the terms. The corresponding class is all the MeSH-C head chapters, corresponding to 22 medical fields: infections, ophthalmologic, stomatology, cardiovascular, digestive, respiratory, nervous, etc. A BERT model for the sequence classification was used and trained on all annotated entities and all MeSH terms and their synonyms. Synonyms of MeSH terms were obtained by extracting all the French terms sharing the same code unique identifier in the unified medical language system defined by their authors as a “set of files and software that brings together many health and biomedical vocabularies to enable interoperability between computer systems” [25]. This multilabel classifier has been described in our previous study and evaluated on an external challenge with an F1-score from 0.809 to 0.811 depending on the model used [18]. For instance, for our index_patient in Figure 1, parietal focal status epilepticus is labelled as nervous, and systemic lupus erythematosus is labelled as immune and skin.

### Distance Computation

We used FastText to obtain an embedding representation of each extracted term. With all the patients represented as a list of embeddings for each label, the distance between the patients can be computed based on one particular label of interest (cardiovascular, urogenital, etc), or several, or all. However, 2 patient records may contain different numbers of terms (ie, vectors) per label. For example, index_patient on Figure 1 only presents 1 term on the cardiovascular label (lupus pericarditis), whereas patient_2 may present many cardiovascular terms such as coronary syndrome, hypertension, and stroke.

Following Kusner et al’s [26] idea, we decided to use the earth mover’s distance, a distance that minimizes the cost to be paid to transform one distribution into another. We compute this distance for each label. In our case, the distributions correspond to the set of terms per label, and each term corresponds to a point. The size of the point corresponds to the frequency of occurrence of the term, and the distance between the points corresponds to the cosine distance between the FastText embeddings of the terms. In our example, the immune label for index_patient is made of the terms SLE (1 occurrence), Raynaud (1 occurrence), Gougerot-Sjögren (1 occurrence), and lupus pericarditis (1 occurrence).

Having a distance, we are now able to compare patients’ clinical notes on each label (provided that the patient’s record has at least one term present for this label) or globally. To compare 2 patients globally, we summed the earth mover’s distances of the 2 patients across each label and weighted them with the corresponding number of terms for each label. Equations (1) and (2) below specify the weighting term, where HR_1_ and HR_2_ denote 2 different hospitalization reports, and EMD() denotes the earth mover’s distance between the 2 notes for a specific label i.


D(HR_1_, HR_2_) = (1/nlabels)*Σ (λ_i_ EMD(HR_1_(label_i_), HR_2_ (label_i_)) **(1)**

with λ_i_ = (nHR_1_(label_i_) + nHR_2_(label_j_)) / (nHR_1_ + nHR_2_) **(2)**

where HR_j_(label_j_) is the list of terms from HR_i_ involving label_j_ and nHR is the number of terms in the term subset HR.

### Evaluation

We evaluate our approach with the 6 use cases described earlier, each being associated with specific MeSH-C labels. For example, to obtain similar patients for the osteoporosis phenotype (labelled musculoskeletal and nutritional according to MeSH classification), we computed the earth mover’s distance of the hospitalization reports only on these 2 labels. Similarly, for ILD in systemic sclerosis, we focused on the respiratory and immune labels. For lung infection, we focused on the respiratory and infections labels, and so on. However, our algorithms can be applied to any new use case and to any set of MeSH-C labels.

#### Clustering

To visualize our results and to confirm the relevance of our approaches, we performed an unsupervised hierarchical clustering of all patients in the training data set on each label and globally, checking if patients with similar phenotypes belonged to the same clusters. We used agglomerative hierarchical clustering (each hospitalization report is initialized as a singleton cluster, and clusters are merged two-by-two) with Ward’s criterion, which minimizes the variance of the clusters. The same method was used for our 6 use case phenotypes. We used the SciPy library [27].

#### Selection of a Cohort of Similar Patients From an Index Patient

**Figure 2:**
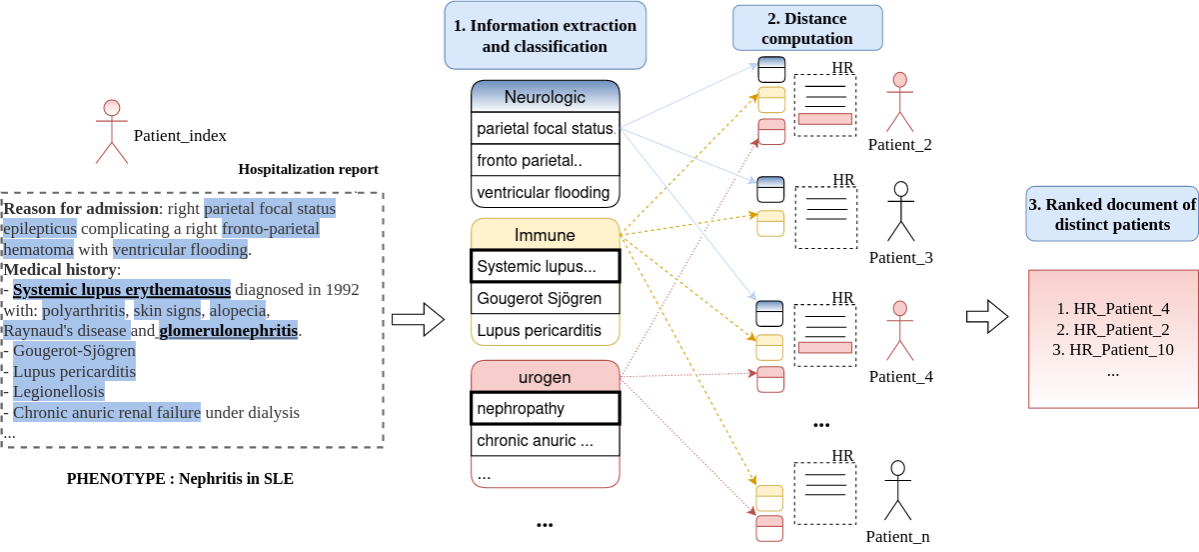
Example of document selection for the phenotype "Nephritis in systemic lupus erythematosus." First, from the clinical observation of the index patient, symptoms and diseases are extracted and classified according to medical subject heading-C chapter headings (step 1). Then, the distance is calculated on the UroGen and immune classes (specifically for this phenotype, step 2). Finally, the closest documents are those with the same written phenotype, corresponding to the patients in red in the figure, leading to a ranked list of the closest documents of distinct patients (step 3). SLE: Systemic lupus erythematosus; HR: Hospitalization report.

We approach the problem of building a cohort of similar patients as an information retrieval problem, where the patient’s document (index patient) is a query. We then evaluate the ability of the system to return a ranked list of documents, with the most relevant/similar at the top of the list. Figure 2 gives an overview of this selection on the example of a patient with the phenotype “Nephritis in SLE.” We evaluate the precision-at-k (percentage of correct phenotype prediction in the first k closest documents of distinct patients), the recall (percentage of all correct phenotypes that are selected in the first n closest patients, n being the number of patients in each phenotype), and the average precision. The average precision computes the average value of the precision for recall values over 0 to 1. It considers the order in which the patients are selected and corresponds to an estimate of the area under the precision-recall curve. For each phenotype, each patient from the test set is chosen in turn as an index patient, and the final results are an average over all patients. Confidence intervals were calculated using the normal distribution approximation.

#### Visualization

A distance-based search result was also constructed to select the most similar patient to an index patient, with clickable labels where clinicians can choose any labels of interest they want to select (as in our phenotype examples). This search result returns the most similar patients on the selected labels in the descending order of similarity. A demonstration can be found in this following link [28], with 4 use cases with word clouds of medical terms enabling the similarity decision. All our codes are available on GitHub [29].

### Ethics Approval

The results shown in this study are derived from the analysis of the AP-HP data warehouse. This study and its experimental protocol was approved by the AP-HP Scientific and Ethical Committee (IRB00011591 decision CSE 20-0093). All methods were carried out in accordance with relevant guidelines (reference methodology MR-004 of the Commission Nationale de l’Informatique et des Libertés [19]). All medical records have been pseudonymized. Patients are informed by the AP-HP data warehouse that the data are pseudonymized and that they can object to their sharing. Their consent was therefore collected prior to our study.

## Results

### Clustering

**Figure 3:**
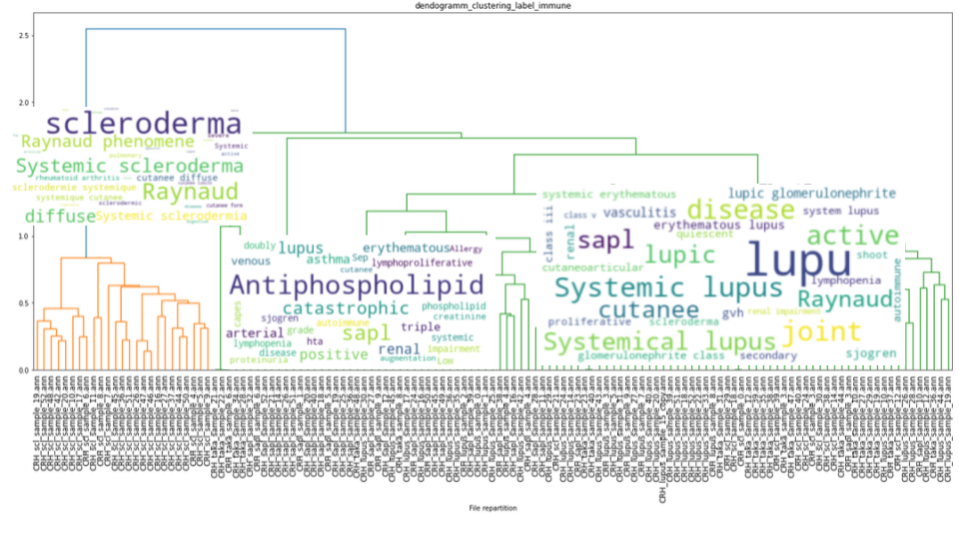
Unsupervised hierarchical clustering based on electronic health record earth mover's distance on the “immune” label. Word clouds of electronic health records words are plotted on each respective cluster. Interestingly, patients with systemic scleroderma all belong to the same cluster (orange). Only patients who were labelled “immune” are clustered; we thus represent 129 patients out of 151.

**Figure 4:**
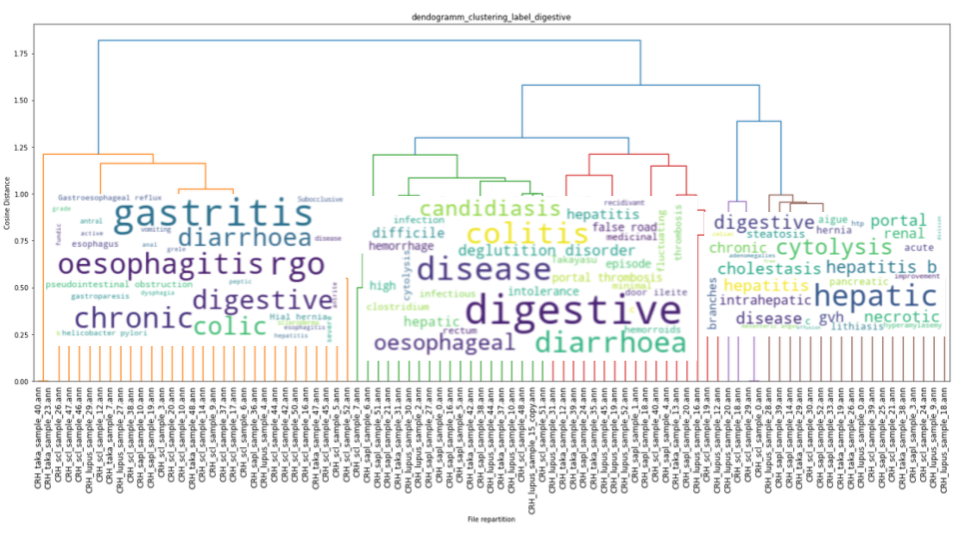
Unsupervised hierarchical clustering based on earth mover's distance of electronic health records on the label “digestive.” The word cloud of the electronic health records is shown on each respective cluster. Interestingly, the left cluster reports upper digestive manifestations (oesophagitis, gastroesophageal reflux or RGO in French), and the rightmost cluster represents patients with liver diseases (brown cluster: cytolysis, hepatitis, hepatic), whereas the middle cluster represents patients with both conditions. Only patients who were labelled digestive are clustered; we thus represent 89 patients out of 151.

**Figure 5:**
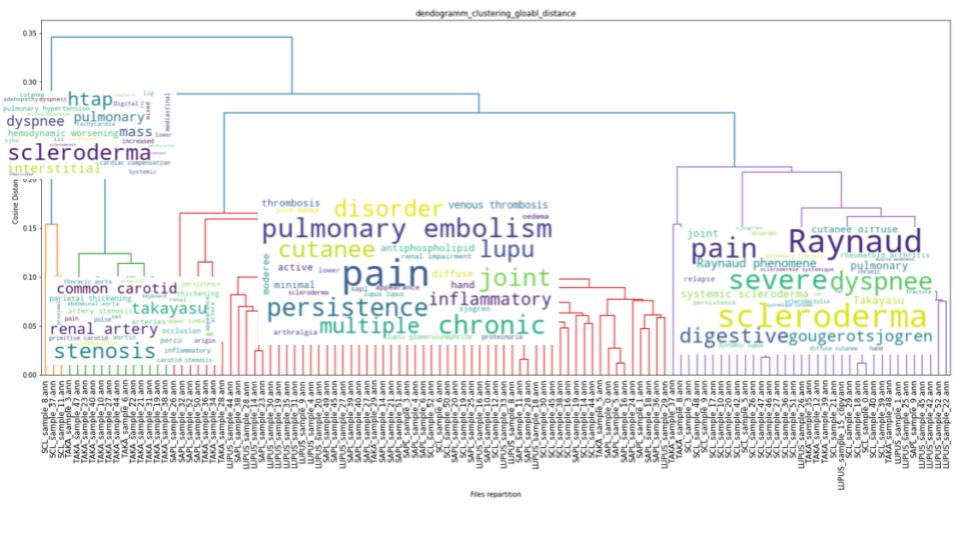
Unsupervised ascending hierarchical clustering based on the overall earth mover's distance of the electronic health records from equations (1) and (2). Word clouds of term frequency in the electronic health records are plotted on each respective cluster.

**Figure 6:**
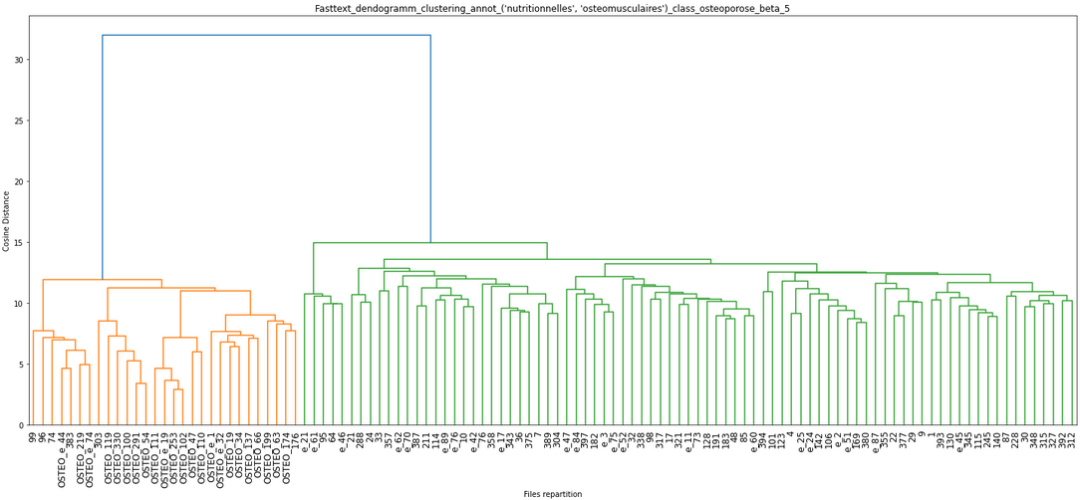
Unsupervised ascending hierarchical clustering based on earth mover's distance of electronic health records on the “osteomuscular” and “nutritional” labels (derived from the medical subject heading classification); only patients having the labels “osteomuscular” and “nutritional” are represented here (corresponding to 119 patients, not 256). All patients with osteoporosis were labelled “OSTEO” in the orange cluster. Other patients present in this cluster without explicitly written osteoporosis present “osteopenia” (all 4 first patients) of several vertebral fractures.

**Figure 7:**
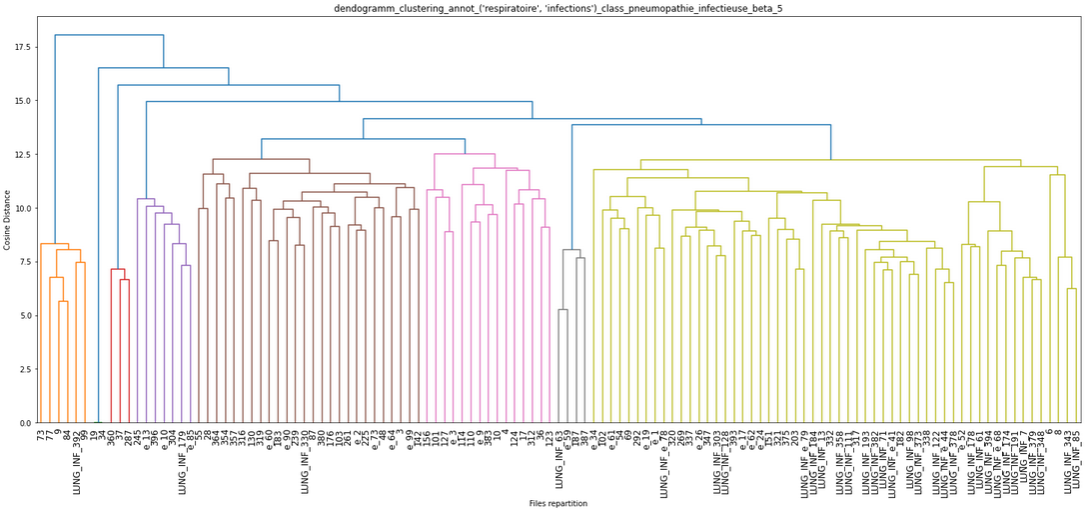
Unsupervised ascending hierarchical clustering based on earth mover's distance of electronic health records on the respiratory and infection axes (derived from the medical subject heading classification). All patients with lung infections were labelled “LUNG_INF” in the green cluster. Some outliers may be noticed; on the very left, the patient had purulent pleurisy, and one had pulmonary tuberculosis. The remaining patients on the left of the green cluster all had other linked manifestations such as bronchitis, parainfluenza infection, and bronchoalveolar lavage positive for *Klebsiella pneumoniae* and oropharyngeal flora.

The results of the unsupervised hierarchical clustering on our training data set of 151 EHRs are shown in Figure 3, Figure 4, and Figure 5. Each cluster is enhanced with its corresponding word cloud (highlighting the frequencies of occurrence of terms within each cluster). Interestingly, on the immune label (Figure 3), we were able to properly separate patients with scleroderma (left, orange cluster) from patients with lupus or lupus with APS (green clusters). As mentioned earlier, 30% of APS is secondary to systemic lupus, and indeed, several patients with APS in our data set also had lupus. Similarly, on the digestive label (Figure 4), we were able to separate upper digestive manifestations (left cluster) from liver issues (left clusters). With regard to the global clustering (using equations 1 and 2 above), we obtained 4 different clusters, as shown in Figure 5. Scleroderma is clustered separately with forms of cutaneous lupus (right, purple cluster) from lupus with thromboembolic manifestations and APS (middle, red cluster) from Takayasu (second left, green cluster). Interestingly, scleroderma with pulmonary arterial hypertension (left, little orange cluster) is close to the Takayasu cluster with arterial complications. The test set included 100 patients with lupus, 87 with scleroderma, 51 with APS, and 18 with Takayasu arteritis. Only 4 Takayasu stroke were labelled and 7 obstetrical APS, which did not allow us to perform clustering or other performance computations. The clustering results for phenotypes osteoporosis and lung infection with ground truth labelled documents are shown as examples in Figure 6 and Figure 7, respectively.

### Selection of a Cohort of Similar Patients From an Index Patient

The performance of cohort construction for the first 4 phenotypes is presented in Table 1. The last 2 phenotypes (P5-P6) could not be analyzed due to a limited number of phenotypes at the annotation stage (7 and 4, respectively).

Overall, we obtained an average precision ranging from 0.58 to 0.88, precision@10 from 0.65 to 0.98, and recall from 0.53 to 0.83. However, the average precision was lower for P3 (ILD in systemic sclerosis) owing to the higher diversity of terms used to describe the lung condition, that is, fibrosis, ILD, scleroderma with pulmonary involvement, etc, and to the fact that the phenotype annotations were very specific. As an example, sclerodermatomyositis or mixed connective tissue disease with lung involvement, which are very close to this phenotype were not annotated positively. An error analysis with mention encountered on close patients can be found in Table S1 of Multimedia Appendix 1. For the 4 phenotypes P1-P4, the precision-recall curves (means for all patients within each phenotype) were computed and are shown in Figure S1 of Multimedia Appendix 1, which is another way of showing the average precision performances. We showed very good results for the P1-P2 and P4 phenotypes and satisfactory results for the P3 phenotype since the patients had to present exactly the same disease.

**Table 1:** Performance results for phenotype similarity (mean and 95% CI) for all patients of a phenotype. For each phenotype, each patient in the test set is chosen in turn as an index patient, and the final results are an average of all patients.

	P1, osteoporosis (n=23)	P2, nephritis in systemic lupus erythematosus (n=48)	P3, interstitial lung disease in systemic sclerosis (n=20)	P4, lung infections (n=33)
Precision@3^a^	0.97 (0.91-1.0)	0.99 (0.98-1.0)	0.85 (0.75-0.95)	0.92 (0.84-0.99)
Precision@10	0.95 (0.91-0.99)	0.98 (0.97-0.99)	0.65 (0.58-0.72)	0.86 (0.81-0.92)
Average precision	0.88 (0.85-0.90)	0.85 (0.83-0.87)	0.58 (0.54-0.62)	0.72 (0.69-0.75)
Recall^b^	0.83 (0.81-0.84)	0.79 (0.77-0.80)	0.53 (0.50-0.55)	0.66 (0.64-0.68)

^a^Precision@3 patients (precision@10) is presented, which represents the obtained precision calculated on the 3 (or 10) patients closest to the index patient (ie, with the minimum distance).

^b^Recall is the recall calculated for all patients to be found with the same phenotype (ie, recall calculated on the 23 closest patients for osteoporosis, the 48 closest patients for nephritis in systemic lupus erythematosus, etc). Precision-recall curves for the 4 phenotypes are shown in Figure S1 of Multimedia Appendix 1.

### Visualization

**Figure 8:**
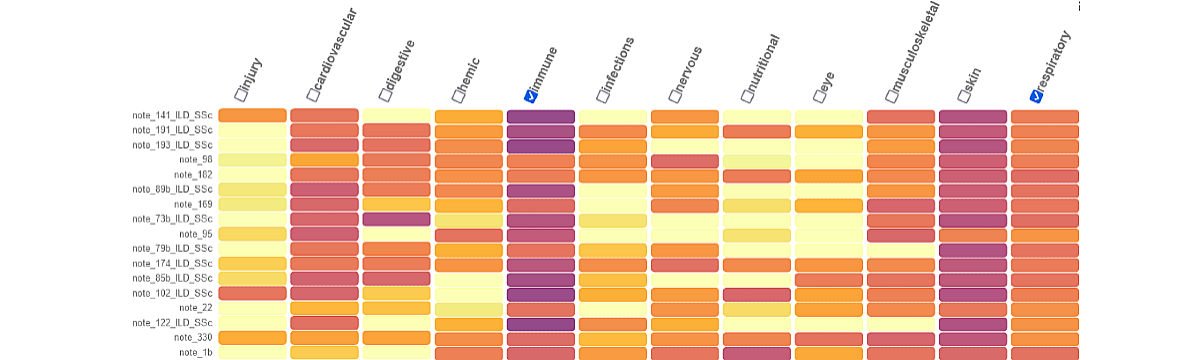
Search results of an index patient with interstitial lung disease; the darker the color is, the closer the patients are to that particular label. Here, the selected labels “immune” and “respiratory” in 8 of the 10 first patients are labelled with “PINS_Sclerodermie” (in French, ie, interstitial lung disease in systemic sclerosis).

**Figure 9:**
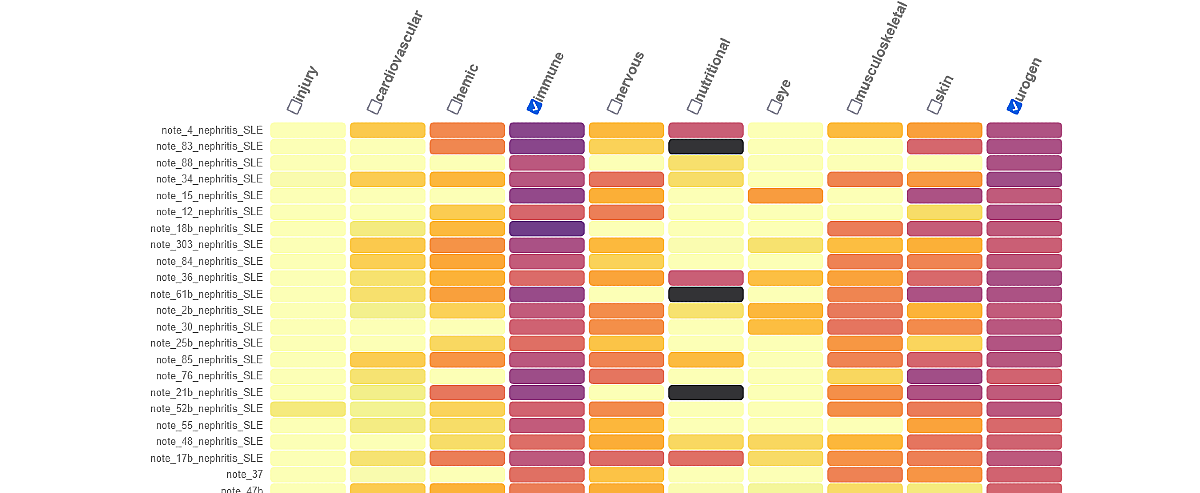
Search results of a patient with nephritis in systemic lupus erythematosus. The darker the color is, the closer the patients are to that particular label. Here, the selected labels “immune” and “urogenital” in all the 20 first closest patients are labelled with the right phenotype nephro_lupus.

As an illustration, Figures 8 and 9 below show the search results described earlier for a patient with ILD in systemic sclerosis and nephritis in SLE, respectively. We see that for an index patient with ILD in systemic sclerosis (Figure 8), choosing the immune and respiratory labels led to the finding of 10 patients out of the 15 first, having the same condition. Interestingly, among these 15 samples, the 5 unlabeled patients had a disease very close to the expected one: “ILD evolving to fibrosis” and a “mixed connective tissue disease” for the first one (note_98, rank 4) and “sclerodermatomyositis” and “interstitial lung disease” for the second (note_182, rank 5). Further analysis of the errors is presented in Table S1 of Multimedia Appendix 1. A more extensive error analysis can be found in Table S1 of Multimedia Appendix 1. Figure 9 shows the search results for an index patient with nephritis in SLE. All the 21st closest patients on labels “immune” and “urogenital” showed nephritis in SLE.

## Discussion

### Summary

In this study, we developed a novel end-to-end algorithm from raw clinical notes to cohort similarity extraction. We have shown that we can cluster very specific phenotypes on an annotated data set and build similarity cohorts with good mean average precision results. These phenotypes and diseases were chosen as a proof of concept, with 2 general phenotypes such as osteoporosis and lung infection and 2 very specific phenotypes with nephritis in SLE and ILD in scleroderma. However, our algorithm can be applied to other phenotypes or diseases as well. Furthermore, our system can be applied to any other data warehouse and does not contain any handcrafted rules. An interactive demo is available online [28], and all our codes are available on GitHub [29].

### Advantages of Our Approach

The main advantage of our approach is the proximity to clinical reasoning—the named-entity recognition step focusing on the distinction between physiological and pathological signs and the observations of the patients on the 22 main medical domains (cardiovascular, pulmonary, hemic, immune)—thereby allowing clinicians to choose on which aspect patients should be similar. This analysis provides interpretable results to clinicians as well as high modularity, which is essential in the field of therapeutic decision support. In clinical practice, this algorithm would enable the physician to automatically extract similar patients, evaluate their clinical evolution, and extrapolate them to the patient they want to treat. Our algorithm focuses on 1 patient’s hospitalization report rather than on the entire patient’s record (EHR), as we want to extract patients with similar conditions and similar acute complications at a time. This algorithm is also able to compare along very fine-grained characteristics. For example, 2 patients with osteoporosis complicated by a bone fracture will be closer than 2 patients with osteoporosis without a fracture. In addition, although our algorithm does not directly consider biological results in a quantitative manner, the clinician’s interpretation of these results in the text is systematically integrated and analyzed as a symptom, for example, anemia, hypoalbuminemia, and positive antibodies. Similarly, the pathological description of imaging reports, such as an alveolar condensation in radiology images or an abnormal left ventricular ejection fraction in echocardiograms will be taken into account in our algorithm. We show very good results in terms of precision and average precision for selecting similar patient cohorts. The robustness of the algorithm is demonstrated on the one hand by the evaluation of the precision-to-3, which is calculated here not for the construction of the cohort but rather to show that there is, as expected, a gradient of similarity from the closest to the most distant patients, and on the other hand, as shown in the error analysis, patients close to a given index patient had very similar disease, even if the exact phenotype was not encountered.

### Comparison With Previous Work

Other studies have focused on patient similarity cohorts; for instance, in the French language, Garcelon et al [30] used a patient representation and a similarity measure to try to find patients with rare diseases in the Dr Warehouse database [31]. Although their objective is quite similar to ours, they used a different representation based on the term frequency–inverse document frequency weights of the extracted concept in each clinical note, and the concept extraction is based on handcrafted rules. They obtained a percentage of 71%-99% of indexed patients returning at least one similar true-positive patient within the first 30 similar patients, and the average number of patients with exactly the same disease among the 30 patients was 51%. In a second study based on the same term frequency–inverse document frequency similarity metric, they evaluated the association between clinical phenotypes and rare disease and measured the relevance of the first 50 similar patients by a domain expert a posteriori; they obtained average precision from 0.55 to 0.91 on 6 phenotypes with mean average precision of 0.79 [32]. The main differences from our method are that we focus on clinical interpretability, and our metric computation is based on one of the most recent and performant language models [12]. Moreover, in our case, the test set was annotated a priori. Jia et al [33] also proposed an interesting algorithm for diagnostic prediction based on patient similarity, but unlike our method, their named-entity recognition step is based on a dictionary of symptoms, while disorders are extracted from ICD-10 coding. The similarity regarding symptoms is binary: 1 if the symptom is shared by both patients and 0 if otherwise. The similarity of diseases is based on their respective ICD-10 similarity (using the ICD-10 coding tree structure).

Ng et al [34] presented an insightful method based on a precision cohort (ie, patient-similarity cohorts) to help clinicians make treatment decisions for chronic diseases. They trained a global similarity model on a set of thousands of predefined variables (disease variables were constructed using their ICD-9 and ICD-10 codes, laboratory variables with their Logical Observation Identifiers Names and Codes, etc) that learns a disease-specific distance (for the 3 chronic diseases presented: hypertension, type 2 diabetes mellitus, and hyperlipidemia), with significant manual work to build the training data set. The authors did not compute direct measures of similarity cohorts but the direct impact of their method, with 75%, 74%, and 85% of decision points in hypertension, diabetes, and hyperlipidemia, respectively, and with at least one significantly better treatment. In contrast, our method focused on the performance of the similarity cohorts with metrics used in the information retrieval field, does not rely on manual variable definition, and does not learn disease-specific distance but a completely generic distance. One of the main advantages of our work is the original calculation of distance per class between patients; to the best of our knowledge, there is no similar work in the literature to compare our work to. However, we show that the named-entity recognition algorithm obtained state-of-the-art results, and the multilabel classification obtained the same performance as the best team of a French national challenge [18].

### Limitations

Our work has several limitations. First, it does not cover mental health diseases, which are a completely different branch of the MeSH classification. However, training the multilabel classifier with a new label for mental health diseases with MeSH terms and synonyms can be done fairly directly based on our framework. In addition, due to time constraints, the data used in this paper were labeled by only 1 internist, and the quality of the data labeling cannot be assessed. In addition, one could argue that we did not compare our clustering and cohort similarity extraction with an ICD-10 extraction. However, because we built our initial data set with ICD-10 codes for our 4 main pathologies, we had an initial bias that we could not overcome for fair comparison. In addition, nephritis in SLE, ILD in systemic sclerosis, and lung infections do not have direct ICD-10 codes used in clinical practice. For example, “glomerular disease with SLE” has the ICD-10 “M3214” but in the entire database of 39 different hospitals, no patient had this particular code. This is because the coding is primarily done to describe the severity of the patient being managed, and this last code, in particular, does not reflect the severity of the renal involvement (in our case, codes for nephritis usually used would be N03, N04, or N05 and M320, M321, M328, and M329 for SLE). Similarly, scleroderma with pulmonary involvement has an ICD-10 code M348 that also does not appear in our database.

Assuming that an important clinical fact is repeated several times in a clinical report (eg, a patient hospitalized for acute coronary syndrome will have many cardiovascular terms linked to his/her cardiac condition), our distance computation from equations 1 and 2 depends on the number of terms in the document. Hence, 2 patients with the same major (repeated) problem would be relatively close. However, sometimes, repeated terms are not directly derived from a major clinical fact (for instance, medical history may be repeated several times without clinical relevance).

### Conclusion

In this work, we have presented a novel end-to-end interpretable algorithm to automatically extract similar patients from an index patient based on clinical note analysis. Our algorithm shows good performance results for 4 specific phenotypes in the context of 4 systemic diseases. In this work, we focused only on pathological signs, but in clinical practice, one could also be interested in negative signs (for instance, the absence of Raynaud syndrome is very atypical in systemic sclerosis). This will be added in our future work, thereby adding a new physiological dimension to patients. In future work, the drug information will also be added for patient comparison, and similar to our presented approach, the clinician will then be able to focus only on treatments or on treatments and signs and symptoms. Finally, we will consider patients as a set of multiple longitudinal hospitalization reports (EHRs). An important perspective of this work is also to evaluate this tool in clinical practice.

## Appendix

Multimedia Appendix 1 Examples of terms extracted from a hospitalization report close to an index patient with interstitial lung disease.

## Abbreviations

AP-HP: Assistance Publique-Hôpitaux de Paris
APS: antiphospholipid syndrome
BERT: Bidirectional Encoder Representations from Transformers
EHR: electronic health record
ICD-9/ICD-10: International Classification of Diseases, Ninth/Tenth Revision
ILD: interstitial lung disease
MeSH: medical subject heading
SLE: systemic lupus erythematosus

## Data Availability

The data sets generated during this study (anonymized similarity measures between patients for the 4 use cases described in this paper) are available in the data repository at this link [35]. The data sets analyzed in this study are not publicly available due the confidentiality of data from patient records, even after deidentification. However, access to the Assistance Publique-Hôpitaux de Paris data warehouse’s raw data can be granted following the process described on its website [36] by contacting the Ethical and Scientific Community at secretariat.cse@aphp.fr. A prior validation of the access by the local institutional review board is required. In the case of researchers who are not from the Assistance Publique-Hôpitaux de Paris, the signature of a collaboration contract is mandatory.
