# Supplementary material for: Construction of Cohorts of Similar Patients From Automatic Extraction of Medical Concepts: Phenotype Extraction Study
Source: JMIR Med Inform. 2022 Dec 19;10(12):e42379. doi: 10.2196/42379 (PMC9808583; doi:10.2196/42379)

**Multimedia Appendix 1**

**Table S1:** Examples of terms extracted from hospitalization report close to an index ILD patient. The rank written describes the distance to the index patient.

| note_98 (rank 4) | “ILD evolving to fibrosis,” “lupus,” “systemic lupus,” ‘stability of the ILD with fibrosis” , “mixted connective tissue disease” |
| --- | --- |
| note_182 (rank 5) | “sclerodermatomyositis,” “interstitial lung disease,” “pulmonary aggravation” |
| note_169 (rank 7) | “raynaud syndrom,” “interstitial lung disease,” “inflammatory myopathy” |
| note_95 (rank 9) | “sharp syndrom,” “mixted connective tissue disease,” “early diffuse interstitial lung disease” |
| note_22 (rank 15) | “lupus,” “gougerot sjogren,” “pulmonary hypertension,” “lupus erythematosus with skin lesion” |
| note_330 (rank 17) | “lupus with lung involvement,” “lung fibrosis,” “secondary gougerot sjogren syndrom” |
| note_1b (rank 18) | “systemic lupus with pulmonary fibrosis,” etc… |
| note_373 (rank 20) | “interstitial lung disease,” “raynaud,” “mixted connective tissue disease,” “lupus,” “sjogren,” “pulmonary hypertension,” “pulmonary fibrosis,” … |

**Figure S1.** Precision-recall curves for the 4 phenotypes.
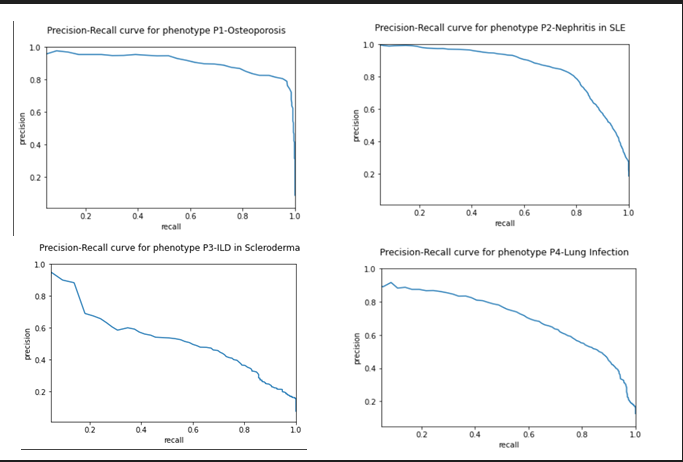

Supplement: Multimedia Appendix 1 [file medinform_v10i12e42379_app1.docx]
